# The link between physician motivation and care

**DOI:** 10.1007/s10198-023-01605-7

**Published:** 2023-06-23

**Authors:** Anne Sophie Oxholm, Dorte Gyrd-Hansen, Christian Bøtcher Jacobsen, Ulrich Thy Jensen, Line Bjørnskov Pedersen

**Affiliations:** 1 Danish Centre for Health Economics, Department of Public Health, University of Southern Denmark, Odense, Denmark; 2 Crown Prince Frederik Center for Public Leadership, Department of Political Science and Government, Aarhus University, Aarhus, Denmark; 3 Center for Organization Research and Design, School of Public Affairs, Arizona State University, Phoenix, USA; 4 Research Unit for General Practice, University of Southern Denmark, Odense, Denmark

**Keywords:** Physicians, Double agency, Financial incentives, Altruism, User orientation, Public service motivation, I10, I11, J23, J24, M50

## Abstract

Studies report an unexplained variation in physicians’ care. This variation may to some extent be explained by differences in their work motivation. However, empirical evidence on the link between physician motivation and care is scarce. We estimate the associations between different types of work motivation and care. Motivation is measured using validated questions from a nation-wide survey of Danish general practices and linked to high-quality register data on their care in 2019. Using a series of regression models, we find that more financially motivated practices generate more fee-for-services per patient, whereas practices characterised by greater altruistic motivation towards the patient serve a larger share of high-need patients and issue more prescriptions for antibiotics per patient. Practices with higher altruism towards society generate lower medication costs per patient and prescribe a higher rate of narrow-spectrum penicillin, thereby reducing the risk of antimicrobial resistance in the population. Together, our results suggest that practices’ motivation is associated with several dimensions of healthcare, and that both their financial motivation and altruism towards patients and society play a role. Policymakers should, therefore, consider targeting all provider motivations when introducing organisational changes and incentive schemes; for example, by paying physicians to adhere to clinical guidelines, while at the same time clearly communicating the guidelines’ value from both a patient and societal perspective.

**Supplementary Information:**

The online version contains supplementary material available at 10.1007/s10198-023-01605-7.

## Introduction

Studies report unexplained variation in physicians’ care and in their responses to policy schemes (e.g. Markovitz and Ryan [1] Cabana, Rand [2]). This variation shows a need for gaining a better understanding of physician behaviour. Physicians’ behaviour may be explained by both supply-side and demand-side factors. One potentially important supply-side factor is physicians’ work motivation. However, empirical evidence on the link between work motivation and care is scarce. In this study, we seek to help address this issue by estimating the associations between different types of work motivation and care.

Physicians’ work motivation is typically explained by their financial motives and altruism towards their patients (e.g. Makris and Siciliani [3], Chalkley [4], Ellis and McGuire [5], Scott [6]). Literature reviews conclude that physicians are *financially motivated* as they respond to financial incentives, although to varying degrees (e.g. Scott, Sivey [7], Jia, Meng [8], Eijkenaar, Emmert [9], Gosden, Forland [10]). Two recent studies support this conclusion. The first study shows that physicians who have a higher marginal utility of income, uncovered by a stated-choice experiment, are more likely to exploit a lack of competition by setting higher prices [11]. The second study uses experimental data and observes that primary care physicians who are more responsive to financial incentives also generate higher profits in reality [12].

A few empirical studies also conclude that *altruism towards patients* affect physicians’ care. These studies make use of different data sources. Laboratory experiments with future physicians verify a willingness to forego own profit for patient benefits (e.g. Hennig-Schmidt, Selten [13], Brosig‐Koch, Hennig‐Schmidt [14], Di Guida, Gyrd‐Hansen [15], Godager and Wiesen [16], Oxholm, Di Guida [17], Attema, Galizzi [18]). Brock, Lange [19] use a combination of experimental and observational data and find that clinicians who are willing to sacrifice own profits on behalf of strangers in an economic game to a higher degree adhere to protocols benefitting the patients in the real world. Douven, Remmerswaal [20] use administrative data to show that providers whose treatment decisions are insensitive to financial compensation obtain better patient outcomes. Jensen and Andersen [21] combine administrative data and survey data and find a positive association between general practitioners’ (GPs) prescribing of antibiotics and user orientation (UO), which describes altruism towards patients.

Some studies also recognise that physicians may be motivated by being *good agents to the third-party payer or society at large* (e.g. Allen, Gyrd-Hansen [22], Blomqvist [23], Oxholm, Gyrd-Hansen [24], Kesternich, Schumacher [25]). In the public administration literature this motivation is called public service motivation (PSM). PSM is defined as an individual’s orientation to delivering service to people with the purpose of doing good for others and society [26]. However, only a few empirical studies investigate the link between PSM and healthcare. Jensen and Andersen [21] find a positive association between GPs’ PSM and the share of narrow-spectrum penicillin prescribed. Prescribing more narrow-spectrum penicillin is in the interest of society as it reduces the risk of antimicrobial resistance compared to other antibiotics. Jensen and Vestergaard [27] find that PSM is positively associated with GPs’ provision of home visits. They assess that home visits are beneficial to the patient and society but poorly remunerated compared to the invested resources. These findings point to the potential importance of also considering altruism towards society when we seek to understand physician behaviour and explain variation in the care provided to patients.

All in all, studies have shown that phycisians respond to incentive schemes, but the empirical evidence on the importance of the underlying motivational profiles for care remains scarce. Such studies are warranted, because different aspects of motivation can have different consequences for phycisian behaviour and care. At the same time, existing studies typically only focus on a narrowly defined spectrum of physicians’ care. This study consolidates and further develops the literature on physician agency by assessing the link between three motivational factors and a large array of real-world indicators of physicians’ care.

Using validated questionnaire items from a nation-wide survey of Danish GPs, we measure physicians’ financial motives (an important component of ‘extrinsic motivation (EM)’), altruism towards the patient (also referred to as ‘user orientation (UO)’), and altruism towards society (also referred to as ‘public service motivation (PSM)’). We link these measures of GP motivation with high-quality register data on general practices’ care. We group our care indicators by *who the practices serve* (the patient composition based on health and socioeconomic statuses of enrolled patients), *how many they serve* (list size per GP and list closure), and *how they serve* (total fee-for-services (FFS) per patient, number of face-to-face consultations per patient, and prescribing behaviour). Using a series of regression models, we estimate the associations between practices’ motivation and care. Because care indicators are provided at the practice level and some practices include more than one GP, we apply several analytical strategies catering to both internal and external validity. Reassuringly, our results are robust across all empirical approaches.

Our findings show that practices with higher EM generate more FFS per patient. Practices with higher UO serve a larger share of high-need patients and also issue more antibiotics perscriptions per patient (a highly demanded service [28]). Practices with higher PSM generate lower medication costs per patient, which may benefit society as these costs are primarily financed by taxpayers in Denmark. They also prescribe a higher rate of narrow-spectrum penicillin, thereby contributing to reducing the risk of antimicrobial resistance in the population. Overall, our findings indicate that policymakers should consider the various provider motivations when introducing organisational changes and incentive schemes. An effective intervention may for example involve paying physicians to adhere to clinical guidelines, while at the same time communicating the guidelines’ value from a patient and a societal perspective.

This study contributes to the literature in several ways. First, the few existing studies on physician motivation typically measure the association between providers’ motivation and care by focusing on single services. We measure the association between providers’ motivations and care for a comprehensive set of measures of care and for several types of motivation. Second, the empirical literature primarily focuses on either financial or non-financial motives of care. These motives may, however, be related such that physicians who are highly altruistic towards the individual patient may also be highly financially motivated and it could be that only one of these motives drive care. A strength of our study is that we can take such potential correlations into account. Third, our study is one of only a few studies using real-world data to measure the direct link between providers’ motivation and care.

The study is organised as follows. Section “A framework for understanding the link between physician motivation and care” presents a framework for understanding the association between physician motivation and care. Section “Institutional setting” introduces the institutional setting for Danish GPs. Section “Data” presents our data. Section “Methods” and Sect. “Results” describe our methods and results, respectively. Section “Disscussion” discusses the results, provides policy recommendations, and outlines scope for future research.

## A framework for understanding the link between physician motivation and care

Health economists typically use agency theory to explain physicians’ motivation to provide care [6, 29]. According to this theory, physicians take on the role of an agent to their patients. Physicians are not always perfect agents as they provide the amount of care that maximises their own utility, which depends on leisure time and income. The health economics literature, however, recognises that physicians also have altruistic concerns, such that they derive utility from being a good agent to the patient [30]. Physicians’ utility maximisation problem is, therefore, typically depicted as [5, 31]:

1 $$\underset{{q}_{\mathit{ji}}}{\mathrm{max}}{u}_{i}\left({q}_{ji}\right)={\gamma }_{i}r\left({q}_{ji}\right)+{\alpha }_{i}{b}^{p}\left({q}_{ji}\right)-{\mu }_{i}c\left({q}_{ji}\right),$$

where ${u}_{i}$ is physician $i$’s utility from providing care, ${q}_{ji}>0$, which may consist of multiple types of care $j$, ${q}_{ji}={q}_{1i},\dots {,q}_{ni}$. Physician remuneration, $r$, is often linked to care, i.e. $r>0, {r}^{\prime}\left({q}_{ji}\right)>0$, and ${r}^{\prime\prime}\left({q}_{ji}\right)=0$. ${b}^{p}$ is patients’ benefit from care, which typically increases with care at a diminishing rate, i.e. ${b}^{p}\left({q}_{ji}\right)>0$, ${b}^{p\prime}\left({q}_{ji}\right)>0$, and ${b}^{p\prime\prime}\left({q}_{ji}\right)<0$, implying that patients do not receive care that decreases their utility and that the first unit of care is the most effective. $c\left({q}_{ji}\right)$ expresses both physicians’ direct costs, such as salaries, equipment, etc., and indirect costs of care in the form of loss of leisure time. These costs increase with care at an increasing rate, i.e. $c\left({q}_{ji}\right)>0$, ${c}^{\prime}\left({q}_{ji}\right)>0$, and ${c}^{\prime\prime}\left({q}_{ji}\right)>0$, capturing the resource constraints physicians face.

A few studies also recognise that physicians may gain utility from being a good agent not only to the individual patient but also to the third-party payer (e.g. Allen, Gyrd-Hansen [22], Blomqvist [23], Oxholm, Gyrd-Hansen [24]). They, therefore, expand the physicians’ utility function with net societal benefits, ${b}^{s}\left({q}_{ji}\right)$. These benefits capture what physicians may consider to be otherwise societal externalities from the provided care:

2 $$\underset{{q}_{\mathit{ji}}}{\mathrm{max}}{u}_{i}\left({q}_{ji}\right)={\gamma }_{i}r\left({q}_{ji}\right)+{\alpha }_{i}{b}^{p}\left({q}_{ji}\right)+{\delta }_{i}{b}^{s}\left({q}_{ji}\right)-{\mu }_{i}c\left({q}_{ji}\right),$$

where we assume that net societal benefits may be either positively, negatively or unrelated to care depending on the type of care provided, ${q}_{j}$. Including net societal benefits in the utility function may help explain why some physicians make treatment decisions not (only) out of concern for the individual patient but from a broader societal perspective.

The standard solution to the utility maximising problem is to choose the amount of care such that the physicians’ marginal benefits equals their marginal costs of care:

3 $${\gamma }_{i}r^{\prime}\left({q}_{ji}\right)+{\alpha }_{i}{b}^{{p}^{\prime}}\left({q}_{ji}\right)+{\delta }_{i}{b}^{{s}^{\prime}}\left({q}_{ji}\right)={\mu }_{i}{c}^{\prime}\left({q}_{ji}\right)$$

This result shows that resource constrained physicians face a trade-off between their costs of care and altruistic benefits when deciding on the amount of care to provide (as shown empirically in e.g. Hennig-Schmidt, Selten [13], Di Guida, Gyrd‐Hansen [15]). The different types of care, ${q}_{1}\dots {q}_{n}$, may impact the components in the utility function to different degrees. In some cases, one type of care yield higher direct and indirect costs, but also generate higher patient and/or societal net benefits than another type of care. In other cases, the choice between different types of care may result in a trade-off between increasing patient benefits and societal net benefits—a so-called double agency problem (e.g. Allen, Gyrd-Hansen [22], Blomqvist [23]). Physicians, therefore, need to decide on the amounts of the different types of care to provide.

As physicians may differ in their relative preferences for remuneration, altruistic concerns, and costs of care, their choice of care may also differ. We express these differences by the relative preference weights, ${\gamma }_{i}\in$[0;1], ${\alpha }_{i}\in [0;1]$, ${\delta }_{i}\in [0;1]$, and ${\mu }_{i}\in [0;1]$. Knowledge about physicians’ preferences enables predictions of their care and thereby also responses to policy schemes. From Eq. (3), we make the following observations about the link between physicians’ preferences and their care,

*Observation 1* If physicians’ preference for financial rewards, ${\gamma }_{i}$, increases, then they will provide more care, ${q}_{j}$, that generates a higher remuneration, $r$.

*Observation 2* If physicians’ altruism towards patients, ${\alpha }_{i}$, increases, then they will provide more care, ${q}_{j}$, that generates higher patient benefits, ${b}^{p}$.

*Observation 3* If physicians’ altruism towards society, ${\delta }_{i}$, increases, then they will provide more care, ${q}_{j}$, that generates higher net societal benefits, ${b}^{s}$.

Our study aims to test the link between practices’ preferences for remuneration, ${\gamma }_{i}$, altruistic concerns, ${\alpha }_{i}$ and ${\delta }_{i}$, and their care, ${q}_{j}$. We proxy practices’ preferences using empirical measures of their work motivation (EM for ${\gamma }_{i}$, UO for ${\alpha }_{i}$, and PSM for ${\delta }_{i}$) and group their care, ${q}_{j}$, by who they serve, how many they serve, and how they serve. We use our theoretical observations to form some expectations about the link between different types of motivation and care (see Sect. “The expected link between motivation and care”).

## Institutional setting

Our case study for investigating the link between physician motivation and care is Danish general practice. General practices play a key role in the Danish healthcare system as they are patients’ first point of contact and gatekeepers for more specialised care. The practices are also responsible for the majority of patients’ preventive care, diagnostics, and treatments [32]. Patients can sign-up with a practice with an open list within close proximity to their home (5 or 15 km depending on the area of location). Practices with more than 1,600 patients on their list per GP can close for further uptake of patients. In 2018, around two-thirds of all practices had closed their list for new patients [33].

In 2019, there were 3365 registered GPs working in 1720 practices in Denmark. Around 25% of these GPs operated in single-handed practices while the remaining 75% operated in partnership practices with two or more GPs. The GPs are self-employed working under a publicly funded contract with the Danish Regions. The Danish Regions pay them a mix of capitation (around one-third of their revenue) and FFS (around two-thirds of their revenue), but do not pay them for their performances. In 2017, GPs’ annual income was on average 1,154,000 DKK (1 DKK = 0.13 EUR) [34]. GPs are not paid for prescribing medication (such as antibiotics), but patients’ uptake of medication is partly subsidised by the taxpayers [35]. For yearly expenses larger than 1020 DKK per patient, the subsidy amounts to 50–100% [36].

## Data

Our measures of work motivation are based on a nation-wide survey of Danish GPs from 2019. The survey includes a number of validated questions on motivation (see Yordanov, Oxholm [37] for an overview of this literature). Some of these questions have also previously been used specifically to measure Danish GPs’ motivation (e.g. Jensen and Andersen [21], Yordanov, Oxholm [37], Pedersen, Andersen [38]). Online resource 1 provides an overview of the questions and their sources. The survey also provides information on practices’ area of location (region and postal code). We use this information to create proxies for structural factors that may affect provision of care, i.e. region and degree of urbanisation. We measure the degree of urbanisation using Statistics Denmark’s definition, which is based on the population density and the number of inhabitants in the largest city in the area [39]. Additional information about the survey is detailed in Yordanov, Oxholm [37].

We measure practices’ characteristics and care using high-quality administrative register data. The Danish Provider Register includes information on whether practices are open for uptake of patients, their number of enlisted patients, the practice type (single-handed or partnership), and whether the practices close during 2019. We measure enlisted patients’ healthcare needs by their health and socioeconomic statuses. We proxy patients’ health status using the Charlson Comorbidity Index [40], which makes use of all registered diagnoses (ICD10-codes) for patients at Danish hospitals from 2015 to 2019. This patient-level information on diagnoses is available from the Danish National Patient Register [41]. As diagnoses are only available from registrations in hospitals, we retrieve a lower bound of patients’ health statuses in general practice. Our measures of socioeconomic status take inspiration from the Danish Deprivation Index [42], which is developed to identify patients in high need of care in general practice in Denmark. We define eight measures of socioeconomic status (see Table 1), which we generate using Statistics Denmark’s registers. We also use Statistics Denmark’s registers to determine whether patients live in Denmark in a given period and the Danish Register of Causes of Death to measure whether patients are alive in a given period [43].

The Danish National Health Service Register includes patient-level data on collected fees from general practices [44]. From this data we obtain information on the practices’ total revenue from FFS as well as the number of face-to-face consultations performed during regular working hours in 2019. From the Danish National Prescription Register we obtain the number, type (the anatomical therapeutic chemical (ATC) codes), prescriber, and costs of redeemed prescriptions at community pharmacies by patients in 2019 [45]. We measure both the total costs of patients’ redeemed medications and the number of redeemed prescriptions of antibiotics issued by the GP.

## Methods

This section describes our empirical strategy. First, we describe our inclusion criteria for practices in our sample. Second, we explain how we generate measures of GP work motivation and describe some potential links between this motivation and our measures of care. Next, we outline how we estimate the link between motivation and care. Finally, we describe our supplementary analyses.

### Study population

Our study population includes general practices that are active the entire year of 2019. We define inactivity as practices that have registered a closing date in the Provider Register during 2019. Our sample of practices is also characterised by having at least one GP responding to the motivational survey. In our analyses, we only include care given to patients who live in Denmark and do not die during a given month in 2019.

### Measuring motivation

We proxy GPs’ preferences for profit and altruism by their EM, UO, and PSM. We measure these work motivations using GPs’ survey responses to a number of validated items measured on a 5-point Likert scale. Online resource 1 provides an overview of these items. As a first step, we use confirmatory factor analyses to investigate whether these items contribute to their latent construct of motivation. Our findings show that the majority of items to a large extent reflect the dimensions of EM, UO, and PSM (see Online resource 2). EM and PSM have a good internal consistency with a Chronbach’s alpha around 0.78–0.79. UO has a low Chronbach’s alpha (0.48), which may be attributed to UO only consisting of three items. These findings are in line with previous studies measuring physicians’ motivation [37]. Similar to previous studies, we keep all items in the main analysis as we believe that they measure important aspects of GP motivation (e.g. Yordanov, Oxholm [37], Pedersen, Andersen [38]).

As a next step, we construct the different measures of motivation. Following other studies on motivation (e.g. Yordanov, Oxholm [37], Pedersen, Andersen [38]), we calculate a simple sum score of the GPs’ responses to the items reflective of each measure. We reduce information loss by mean imputing responses ‘do not know/not relevant’ (EM = 1.2%, UO = 0.5%, PSM = 3.4%). To make it easier to understand the variation within each motivational component, we rescale the sum score to 0–1 using a min–max scaling approach [46], such that a score of zero reflects the least motivated and a score of one reflects the most motivated. As we perform our analysis at practice level (see Sect. “The expected link between motivation and care”), we calculate the average motivation across GPs responding to the survey within a practice. The motivation at practice level is, therefore, more precisely measured for single-handed practices than for partnership practices, where not all GPs may have responded to the survey and where motivation is averaged across respondent. Section “Empirical strategy” outlines several robustness checks to our measures of motivation.

### The expected link between motivation and care

We consider core dimensions of care in general practice, i.e. who they serve, how many they serve, and how they serve. For each dimension we include indicators that broadly capture these dimensions and that are available in administrative registers (see Table 1 for a full list of care indicators). As the chosen care indicators can impact remuneration, patient benefits, or societal benefits, we expect them to be associated with GPs’ preferences for profit and altruism (see observations 1 through 3), proxied by EM, UO, and PSM. Each care indicator may link with more than one type of motivation. In the following, we describe some examples of potential links between care indicators and measures of motivation, but other potential links may also exist.

We measure *who they serve* using ten indicators of high-need patients (see Table 1 and Sect. “Data” for a description). If practices enlist a larger share of high-need patients (proxied by their health and socio economic statuses), then their care will likely generate higher patient benefits and/or net societal benefits, depending on how the indicator reflects individual patients’ benefits and/or otherwise societal externalities. We, therefore, expect a positive association between serving high-need patients and UO and/or PSM. We measure *how many they serve* using two indicators of list size. As part of Danish practices’ remuneration is capitation-based, they have a financial incentive to enlist patients. We, therefore, expect that list size is positively associated with EM, but it could also be positively linked with altruistic concerns so as to ensure patient access to a care. We measure *how they serve* using five indicators of their services and prescribing. As Danish practices’ are also paid by FFS, they have a financial incentive to provide services to patients. We, therefore, expect that practices’ generation of FFS (and consultations linked with a fee) is positively associated with EM. We cover practices’ prescribing patterns using the case of antibiotics. This case is interesting because practices may face a double agency problem and because the chosen indicators proxy quality of care as they relate to clinical guidelines. Studies find that patients often demand antibiotics [28], while society discourages its use due to the risk of antimicrobial resistance in the population [47, 48]. We, therefore, expect a positive association between number of anbiotics prescriptions and UO, whereas we expect a negative association with PSM. To reduce the risk of antimicrobial resistance the clinical guidelines recommend prescribing of narrow-spectrum pencillins instead of broad-spectrum antibiotics. Meanwhile, prescribing of narrow-spectrum pencillins requires additional testing and is less convenient for the patient [22]. We, therefore, expect a negative association between the share of narrow-spectrum penicillin prescribed and UO, whereas we expect a positive association with PSM.

### Empirical strategy

As care is measured for general practices, we perform the analysis at practice level. First, we estimate the association between practice $k$’s motivation and who they serve, i.e. the share of high-need patients on their list, ${\varvec{p}}$:

4 $${p}_{k}={\beta }_{1}{\overline{em} }_{k}+{\beta }_{2}{\overline{uo} }_{k}+{\beta }_{3}{\overline{psm} }_{k}+{e}_{k},$$

where $\overline{em }$ (an indicator of the relative preference weight $\gamma$) measures the practice’s average degree of financial motivation, $\overline{uo }$ (an indicator of $\alpha$) measures the practice’s average degree of altruism towards the patient, and $\overline{psm }$ (an indicator of $\delta$) measures the practice’s average degree of altruism towards society. As we wish to estimate the link between each type of motivation and care, independently of the other motivational factors, we include all motivational components in the same regression. We thereby avoid potential issues with, for example, PSM being related to UO when estimating net societal benefits of care.

Second, we estimate the association between practice $k$’s motivation and how many patients they serve, ${\varvec{l}}$. We measure the practice’s list size by how many patients are enrolled per GP, such that our findings are not explained by practice size:

5 $${l}_{k}={{\vartheta }_{1}\overline{em} }_{k}+{\vartheta }_{2}{\overline{uo} }_{k}+{\vartheta }_{3}{\overline{psm} }_{k}+{\varepsilon }_{k }$$

We also investigate the degree to which this link between motivation and list size is driven by who the practice serves, i.e. their share of high-need patients on their list, ${\varvec{p}}$:

6 $${l}_{k}={\tau }_{1}{\overline{em} }_{k}+{\tau }_{2}{\overline{uo} }_{k}+{\tau }_{3}{\overline{psm} }_{k}+{{\tau }_{4}{\varvec{p}}}_{{\varvec{k}}}+{u}_{k}$$

We further try to understand this link between motivation and how many patients are served by estimating the link between motivation and having a closed patient list (see Sect. “Institutional setting” for a description of this institutional feature). We, therefore, also estimate Eqs. (5), (6) using an outcome dummy for list closure.

Third, we estimate the association between practice $k$’s motivation and how it serves, i.e. the average services per enlisted patient, ${\varvec{s}}$. We focus on core services, i.e. FFS generated per patient, number of face-to-face consultations per patient (which is linked to a fee), and costs of prescription medication per patient. We also include two quality measures related to prescribing of antibiotics, i.e. measures that are related to clinical guidelines:

7 $${s}_{k}={\theta }_{1}{\overline{em} }_{k}+{\theta }_{2}{\overline{uo} }_{k}+{\theta }_{3}{\overline{psm} }_{k}+{\epsilon }_{k}$$

We further investigate the degree to which these associations between motivation and how the practices serve are driven by who they serve, ${\varvec{p}}$, and how many they serve, $l$:

8 $${s}_{k}={\rho }_{1}{\overline{em} }_{k}+{\rho }_{2}{\overline{uo} }_{k}+{\rho }_{3}{\overline{psm} }_{k}+{\rho }_{4}{{\varvec{p}}}_{{\varvec{k}}}+{\rho }_{5}{l}_{k}+{\omega }_{k}$$

We estimate all regressions [4–8] using OLS with robust standard errors. The exception being the outcome of list closure, where we use a binary logit regression with robust standard errors and report the estimates as odds ratios. We investigate issues with multicollinearity by calculating the variance inflation factors (VIF scores). Our estimates of interest are ${\widehat{\beta }}_{1}$-${\widehat{\beta }}_{3}$, ${\widehat{\vartheta }}_{1}$-${\widehat{\vartheta }}_{3}$, ${\widehat{\tau }}_{1}$-${\widehat{\tau }}_{3}$, ${\widehat{\theta }}_{1}$-${\widehat{\theta }}_{3}$, and ${\widehat{\rho }}_{1}$-${\widehat{\rho }}_{3}$, which measure the association between the different types of motivation and the different types of care.

We perform several supplementary analyses to check the robustness of our results. First, we check whether our choice of how to measure motivation affects our results. We use the weights from the confirmatory factor analyses to calculate sum scores, exclude ‘do not know/not relevant’ responses, and use dichotomised measures of motivation (cut-off at both the 50 percentile and 75 percentile of the motivation score distributions) to account for a potential non-linear relationship between motivation and care. Second, we investigate whether the link between practices and GPs affects our findings. Our analysis is performed at practice level, such that we average the motivational scores within a practice. As the average number of GPs per practice is 2.5, we do not believe that the lack of a direct link between motivation and care is a large issue. However, as a robustness check we only consider singlehanded practices where the link is stronger. Third, we investigate whether the inclusion of additional explanatory variables affect our findings. This would be the case, if our regression models suffer from omitted variable bias. Examples of omitted variables may be structural factors, such as access to and collaborations with other providers, both affecting motivation and care. In a supplementary analysis, we, therefore, control for observable structural factors measured as the degree of urbanisation and the region where the practices are located. Lastly, we investigate whether the link between motivation and care is better explained if we instead of only entering all the motivations as independent explanatory variables in the linear regression also include interaction terms between them.

## Results

### Descriptive statistics

Our sample consists of 795 practices corresponding to 50% of the study population. We find that these practices differ from the study population on some of the observable characteristics and outcomes (see Online resource 3). Our sample of practices are less often single-handed practices, have slightly fewer patients with a low socioeconomic status, have fewer patients on their list (59 less on average), and generate a higher activity (FFS) per patient (an additional DKK 27 on average). They are also to a larger extent located in a rural area, which is reflected by them being less present in the Capital Region of Denmark and instead more present in the Region of Southern Denmark and the Central Denmark Region.

**Fig. 1:**
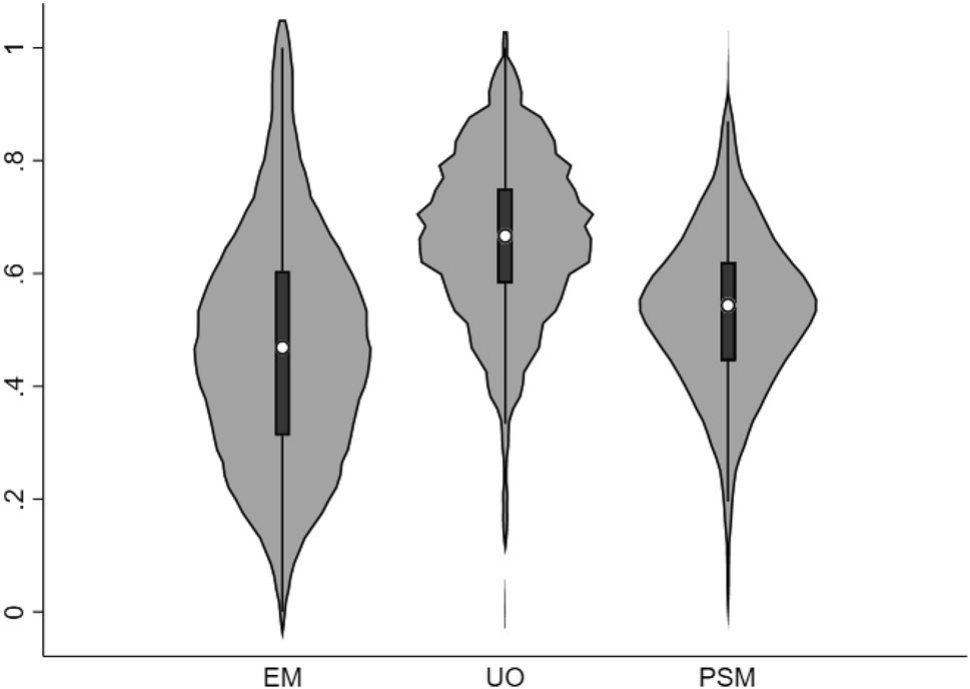
Standardised scores of motivations at practice level. The violin plots are a modification of box plots that add plots of the estimated kernel density. The white dots indicate the medians, the black boxes indicate the interquartile ranges, and the black lines stretched from the bars indicate the lower/upper adjacent values. By definition, none of the observations are below 0 or above 1

Figure 1 shows the distribution of our three measures of motivation across all practices. We find that the variation within each motivational component follow fairly symmetrical distributions. Because the scores are a result of the framing of the survey responses, we cannot compare them across motivational components. Online resource 4 shows that the measures of motivation are not highly correlated. The low correlation suggests that the measures generally do not explain the same type of motivation. For partnership practices (around 69% of practices) the motivational scores reflect an average across responding GPs (see Sect. “Measuring motivation”). Around 44% of the partnership practices have responses from more than one GP with a within-practice variation (average standard deviation) of EM = 0.17, UO = 0.12, and PSM = 0.12.

**Table 1:** Descriptive statistics^a^

2019 (practice level)	Mean	SD
Who they serve?		
Share of enlisted patients who are/have		
20–59 year olds and unemployed for at least 6 months	0.008	0.004
25–59 year olds without vocational education	0.107	0.025
25–65 year olds with low disposable family income	0.122	0.046
18–59 year olds on welfare payments	0.061	0.021
0–16 year olds in family with low educational level	0.011	0.006
Immigrants and descendants from non-Western countries	0.082	0.075
+ 30 year olds who are single	0.153	0.032
+ 70 year olds with a low disposable family income	0.037	0.021
Charlson’s Comorbidity Index equal to 1 (ill patients)	0.037	0.009
Charlson’s Comorbidity Index greater than 1 (severely ill patients)	0.050	0.013
How many they serve?		
List size per GP in the practice	1659	454
Whether the practice operates with a closed list	0.653	0.476
How they serve?		
Services provided by the practice during regular working hours to enlisted patients		
FFS per enlisted patient (DKK)	888	153
Number of face-to-face consultations per enlisted patient	2.94	0.50
Prescriptions redeemed by enlisted patients		
Costs of all prescriptions per enlisted patient (DKK)	1465	301
Number of antibiotic prescriptions issued by the GP per enlisted patient	0.273	0.085
Share of narrow-spectrum penicillin issued by the GP to enlisted patients	0.270	0.059
Number of practices	795	
Average number of GPs per practice	2.48	

^a^This table shows descriptives statistics for our measures of practices’ care. Costs are calculated in Danish kroners, where 1 DKK is equal to 0.13 EUR. Antibiotics are identified by the ATC-codes J01 and P01AB0, where narrow-spectrum penicillin is identified by the ATC-code J01CE

Table 1 shows the descriptive statistics for our measures of practices’ care. We measure who the practices’ serve using ten indicators of high-need patients. We observe that the average share of enrolled patients who fulfil the high-need criteria varies across indicators, with the largest share being + 30 year olds who are single (around 15% of enrolled patients). We further find that on average around 9% of the enlisted patients have a Charlson’s Comoribidity Index score of at least one, indicating the presence of one or more chronic conditions.

Regarding how many patients the practices serve, we find that the average list size per GP is slightly above the minimum requirement for list closure, i.e. 1600 patients per GP. We further observe that almost two-thirds of the practices operate with a closed list at the start of 2019, such that they do not enrol new patients. With respect to how practices serve during regular working hours, we find that on average enlisted patients receive three face-to-face consultations in 2019. FFS per patient during regular working hours exceeds the costs of these consultations (DKK 143 per face-to-face consultation, 1DKK = 0.13 EUR) with more than the double amount, showing that practices provide other services on the fee schedule. Table 1 also reports the costs of all prescription medications redeemed per patient as well as the number of prescriptions of antibiotics issued by the GP per patient. Around 27% of redeemed antiobitics prescribed by the GPs is narrow-spectrum penicillin.

### The link between motivation and care

We now turn to our regression analyses to uncover the associations between practices’ motivation and care. Table 2 shows the associations between practice motivation and who they serve. EM is by and large not statistically significantly associated with serving more high-need patients. UO seems to be associated with serving a larger share of high-need patients on some indicators. We observe the most highly statistically significant association between being altruistic towards patients and serving severely ill patients. More specifically, going from being the least to the most altruistic towards patients is associated with serving a 1.1 percentage points larger share of the severely ill patients (22% of the mean). PSM is not statistically significantly associated with serving a larger share of high-need patients.

**Table 2:** The link between practice motivation and who they serve^a^

Outcome	EM	UO	PSM
Share of enlisted patients who are/have			
20–59 year olds and unemployed for at least 6 months	0.0003 (0.001)	− 0.001 (0.001)	0.001 (0.001)
25–59 year olds without vocational education	− 0.002 (0.005)	0.013 (0.007)	0.003 (0.007)
25–65 year olds with low disposable family income	0.0001 (0.008)	0.004 (0.013)	0.010 (0.012)
18–59 year olds on welfare payments	− 0.003 (0.004)	0.009 (0.006)	0.001 (0.006)
0–16 year olds in family with low educational level	− 0.002 (0.001)	0.003 (0.002)	0.001 (0.002)
Immigrants and descendants from non-Western countries	− 0.003 (0.014)	− 0.002 (0.020)	0.020 (0.024)
+ 30 year olds who are single	− 0.002 (0.005)	0.018* (0.008)	0.016 (0.009)
+ 70 year olds with a low disposable family income	− 0.008* (0.004)	0.011* (0.005)	− 0.005 (0.006)
Charlson’s comorbidity Index equal to 1 (ill patients)	− 0.002 (0.001)	0.003 (0.002)	− 0.002 (0.002)
Charlson’s comorbidity Index greater than 1 (severely ill patients)	− 0.002 (0.002)	0.011*** (0.003)	0.002 (0.003)
Number of observations (practices)	795		

^a^This table shows estimates of regressions between practice motivation and care (share of high-need patients). Estimates are based on ordinary least square regressions with robust standard errors. Standard errors are in parentheses

*p < 0.05

**p < 0.01

***p < 0.001

Table 3 shows the associations between practice motivation and how many patients the practices serve. We find that practice motivation is not statistically significantly associated with the list size per GP when taking into account who they serve. Practice motivation also does not seem to predict the likelihood of closing a practice list.

**Table 3:** The link between practice motivation and how many they serve^a^

	EM		UO		PSM	
Outcome	(1)	(2)	(1)	(2)	(1)	(2)
List size per GP in the practice	146.3* (72.92)	131.0 (70.06)	82.6 (117.5)	71.4 (110.0)	− 180.7 (115.0)	− 217.2 (123.2)
Whether the practice operate with a closed list	1.081 (0.390)	0.938 (0.363)	0.467 (0.239)	0.805 (0.454)	1.135 (0.600)	0.975 (0.578)
Number of observations (practices)	795					

^a^This table shows estimates of regressions between practice motivation and care (list size per GP/closed list). Estimates for ‘list size per GP’ are based on ordinary least square regressions with robust standard errors. Estimates for ‘whether the practice has a closed list’ are based on a binary logit regression with robust standard errors and are reported as odds ratios. The column numbers express the included controls: (1): no controls, (2): control for who the practices serve (see Table 1 for an overview of included variables). Standard errors are in parentheses

*p < 0.05

**p < 0.01

***p < 0.001

Table 4 shows the associations between practice motivation and how the practices serve their patients. EM is statistically significantly associated with generating a higher FFS per patient. This finding also holds when taking into account how many and who the practices serve. A change from the least to the most extrinsically motivated practice is associated with an increase in FFS per patient of 68–88 DKK (8–10% of the mean). The higher FFS is also reflected by extrinsically motivated practices providing more consultations per patient. Interestingly, the average increase in face-to-face consultations of 0.16 per patient when going from being the least to most financially motivated does not fully explain the average increase in FFS $(0.16\bullet 143\mathrm{DKK}=23\mathrm{DKK versus }68-88\mathrm{DKK})$. This difference indicates that extrinsically motivated practices also to a higher degree provide other types of services.

**Table 4:** The link between practice motivation and how they serve^a^

	EM		UO		PSM	
Outcome	(1)	(2)	(1)	(2)	(1)	(2)
FFS per enlisted patient (DKK)	68.32** (26.33)	87.73*** (21.74)	59.53 (36.07)	13.57 (31.56)	− 34.99 (42.54)	− 7.80 (38.73)
Number of face-to-face consultations per enlisted patient	0.088 (0.094)	0.158* (0.079)	0.206 (0.128)	0.026 (0.113)	− 0.069 (0.142)	0.011 (0.131)
Costs of all prescriptions redeemed per enlisted patient (DKK)	− 70.64 (55.67)	− 10.34 (30.52)	239.9** (75.08)	23.20 (38.27)	− 140.8 (79.38)	− 144.9*** (42.86)
Number of antibiotic prescriptions issued by the GP per enlisted patient	− 0.007 (0.016)	0.008 (0.013)	0.072*** (0.020)	0.034* (0.017)	− 0.008 (0.023)	0.008 (0.019)
Share of narrow-spectrum penicillin issued by the GP to enlisted patients	− 0.001 (0.010)	− 0.00004 (0.010)	0.011 (0.015)	0.014 (0.014)	0.028 (0.017)	0.033* (0.016)
Number of observations (practices)	795					

^a^This table shows estimates of regressions between practice motivation and care (services and costs per patient). Estimates are based on ordinary least square regressions with robust standard errors. The column numbers express the included controls: (1): no controls, (2): control for who they serve and how many they serve (see Table 1 for an overview of included variables). Standard errors are in parentheses

^*^p < 0.05

**p < 0.01

***p < 0.001

Altruistic motivations are not statistically significantly associated with FFS per patient. However, PSM is associated with lower total medication costs per patient, when taking into account who and how many patients the practices serve. Going from being the least to the most altruistic towards society is associated with a decrease in medication costs per patient of 145 DKK (10% of the mean). We also find an association between practices’ altruistic motivations and quality of care. We find that UO is associated with issuing 0.03–0.07 more prescriptions of antibiotics per patient (12–26% of the mean), even when taking into account who and how many patients that are served. PSM is associated with prescribing a larger share of narrow-spectrum penicillin. Going from being the least to the most altruistic towards society increases the share of prescribed narrow-spectrum penicillin by 3.3 percentage points (12% of the mean) when taking into account who and how many patients that are served.

### Supplementary analyses

Online resource 5–10 report the results of our supplementary analyses. These results confirm that our findings to a large extent are robust to how we measure motivation (Online resource 5–8), our link between practices and GPs (Online resource 9), and controlling for structural factors measured as the degree of urbanisation and region of location (Online resource 10). There are, however, exceptions that we wish to highlight. Using dichotomised measures of motivation shows that the choice of cut-off of the motivation scores (50 or 75 percentile of the sample distribution) impacts the statistical significance (but not the sign of the estimates) of a few of our findings. For example, the estimates of the association between PSM and medication costs. Excluding motivational responses ‘do not know/not relevant’ removes the statistical significance of our finding for the association between PSM and the share of narrow-spectrum penicillin prescribed (but again not the sign of the estimate). The lack of statistical significance may be due to a smaller sample size. Similarly, only considering single-handed practices (a much smaller sample) reduces the statistical significance of several estimates, but generally not the estimates’ size and sign. Lastly, we find limited impact of also including interaction terms between the motivational components. Across our linear regression models, only two out of 96 interaction terms are statistically significant. These estimates are available upon request.

## Discussion

A key question for policymakers is why variation in physicians’ care exists. Part of the answer may be that physicians differ in their motivational profiles. Empirical evidence is, however, scarce on the link between physicians’ motivation and care (e.g. Scott, Sivey [7], Jia, Meng [8]). Our study contributes to the literature using real-world data to estimate the association between general practices’ motivation and care. We focus on motivational components that proxy the preferences typically modelled in theoretical studies on physicians’ behaviour (e.g. Ellis and McGuire [5], Allen, Gyrd-Hansen [22], Blomqvist [23]). We make use of a broad set of care indicators capturing different dimensions of care, i.e. who they serve, how many they serve, and how they serve. Our study finds that both financial and altruistic motives are heterogeneous across practices and are associated with care.

We find that physicians’ EM is associated with generating more consultations and total FFS per patient. A behavioural pattern which in a Danish setting, where GPs are remunerated on the basis of the services they provide, leads to an increase in remuneration. The higher activity level does not necessarily imply that extrinsically motivated practices overprovide care. In fact, studies show that in settings with resource constraints, providers’ EM may protect against underprovision of care [15]. Currently, many healthcare systems (including the Danish one) suffer from a shortage of GPs [49], indicating that practices’ EM may be beneficial from a policymaking perspective. However, whether practices’ EM is beneficial depends on the design of the remuneration scheme as it may impact both the quantity, quality, and distribution of care across patients, services, and geographical areas [7, 9, 50]. Our assessment of the consequences of EM on how practices' serve is also limited by the administrative registers, which (apart from prescribing) only capture services linked with a fee.

Physicians’ altruistic motives (UO and PSM) may help ensure an equitable delivery of high-quality care. We find that UO is associated with care. More specifically, practices with high degrees of altruism towards the patients serve a larger share of high-need patients (especially severely ill patients) and prescribe more antibiotics per patient, also when controlling for their patients’ needs. The finding that patient-altruistic practices prescribe more antibiotics per patient may be explained by patient demand for this particular kind of medication [28], whereas the finding that patient-altruistic practices serve a larger share of high-need patients may partly be explained by selection. In Denmark, practices cannot select their patients, but patient-altruistic practices may be better at attracting high-need patients. GPs who are more patient-altruistic may also choose to work in practices located in areas where more high-need patients are served. As GPs are owners of their practices, this sorting typically happens in the beginning of their career. The sorting may be explained by GP characteristics associated with UO, such as GPs’ socioeconomic background, place of birth, etc., which is information we do not have access to. A supplementary explanation may be that GPs, who are user oriented, are more diligent at diagnosing their patients, thus identifying more high-need patients. Finally, we cannot exclude the possibility that GPs may become more patient-altruistic because they face more patients who are in high need of care (reversed causality). Importantly, our findings are aligned with the literature on physician agency. Godager and Wiesen [16] and Douven, Remmerswaal [20] also find heterogeneity in physicians’ patient-altruistic motivations, and Jensen and Andersen [21] observe a positive association between UO and prescriptions of highly demanded medications.

Our results show that PSM is associated with lower medication costs per patient and a higher share of narrow-spectrum penicillin prescribed by the GP. This finding may be explained by practices with high PSM being better at adjusting their patients’ medications, such that they are prescribed fewer and cheaper medications. Another explanation could be that their patients to a lesser extent are being referred to other providers who prescribe medicine. In Denmark, taxpayers finance a significant proportion of the costs of prescription medication. Lower medication costs is, therefore, generally in the interest of society. The type of antibiotics prescribed is also important from a societal perspective over and beyond the individual patient’s interests. Consumption of narrow-spectrum penicillin is less likely to lead to antimicrobial resistance in the population, which is considered one of the largest threats to human health [47, 48]. Physicians who are not driven by PSM may choose to prescribe other types of antibiotics because they require less testing (effort) and they are more convenient for the individual patient [22]. This finding is supported by a previous study by Jensen and Andersen [21] on GPs’ prescribing patterns.

All in all, our results support that financial and altruistic motivations are associated with care. We thereby corroborate the relevance of including such factors as components in the physicians’ utility function. Other studies have also shown that profit and patient benefits are associated with physicians’ behaviour via their responses to financial incentives (e.g. Godager and Wiesen [16], Krasnik, Groenewegen [51]). However, the number of studies showing the direct association between providers’ motivational profiles and healthcare using real-world data is limited. Importantly, we also find that altruism towards society may play a role for care. This finding is aligned with Jensen and Andersen [21], who also show that public service motivated GPs prescribe a larger share of narrow-spectrum antibiotics. Expanding physicians’ utility function with altruism towards society, therefore, appears relevant.

Our study faces some limitations. An important limitation is that although we demonstrate expected associations between practice motivation and provision of care, we are unable to provide unequivocal evidence of a causal relationship between practice motivation and care. Our analyses may suffer from confounders, such as structural factors impacting access to and collaborations with other providers. These factors may both affect practice motivation and care. Importantly and reassuringly, a supplementary analysis shows that our results are robust to controlling for the practices’ region and degree of urbanisation, which are related to such structural factors. Reversed causality may also present challenges to our interpretations. For example, physicians providing care to many high-need patients may over time become more patient-altruistic. Although we do not believe this causal pathway to be dominant, it is nevertheless important to disentangle the causal relationship between motivation and care in future studies.

Another limitation is that we can only measure care at the practice level. To address this methodogical limitation, we use different approaches catering to either the internal or external validity of our findings. In our main analysis, we estimate the association between practices’ average level of motivation and care. This approach caters to the external validity of our findings as we include all practices that have responded to the survey in the analysis. As the average number of GPs in a practice is around 2.5, we do not believe that the lack of a direct link between motivation and care is a large issue. However, as a robustness check we perform the analyses only for single-handed practices. This approach caters to the internal validity of our findings as there is a direct link between physicians’ motivation and care. Reassuringly, the signs and size of the key estimates are similar. However, as expected, the statistical significance in some cases drops due to the smaller sample size.

A further limitation is that our sample is based on practices, where at least one GP has responded to the survey. We find that this sample differs from the study population of general practices in Denmark on some outcomes and observable characteristics, including the share of single-handed practices, the degree of urbanisation, and region. However, as the sample of single-handed practices generate results aligned with the results for the full sample, and as our results are robust to controlling for the degree of urbanisation and region, we do not believe that these differences undermine the generalisability of our findings at a national level. The link between motivation and care may, however, differ for physicians located in other settings and contexts. More research is, therefore, needed to determine the generalisability of our results.

The fact that we observe a link between motivation and care across physicians suggest that not only do the preference weights $\gamma ,$ $\alpha ,$ and $\delta$ differ across physicians, but they may also explain physicians’ utility maximising behaviour. These results indicate that a greater insight into physicians’ utility functions may be beneficial for policymakers. Policymakers may stimulate different activities depending on which type of motivation they cater to. EM is associated with the provision of more remunerated services to patients, UO is associated with serving more high-need patients and providing more prescriptions of antibiotics, and PSM is associated with providing higher quality of care and low-cost care. These findings are all in line with our *ex ante* theory based observations, which lends further credibility to our empirical results. We perceive our study to be an important step towards understanding the link between physicians’ motivations and the care they provide. Naturally, more research is required to verify the causal pathway between motivation and different types of care, and to assess whether motivations are stable across time and settings. Future research may also consider how providers’ motivation impact other behavioural patterns such as, for example, their participation in postgraduate medical education, networking activities, and investments.

### Supplementary Information

Below is the link to the electronic supplementary material.

Supplementary file1 (PDF 235 kb)

## Data Availability

The data used in this study is from pseudoanonymised survey and register data held on a secure server by Statistics Denmark. The data is confidential and person identifiable. We are therefore unable to make any of the data publicly available. All analyses were conducted using Stata. The code used remains on the secure server where all the data is stored.
