# Supplementary material for: The link between physician motivation and care
Source: Eur J Health Econ. 2023 Jun 23;25(3):525–37. doi: 10.1007/s10198-023-01605-7 (PMC10972924; doi:10.1007/s10198-023-01605-7)
Supplement: Supplementary file 1 — Supplementary file1 (PDF 235 kb) [file 10198_2023_1605_MOESM1_ESM.pdf]

Online resource 1

Table 1 Overview of items used to measure work motivation

**Motivational component**
**Extrinsic motivation** (Kuvaas, Buch, Weibel, Dysvik, and Nerstad (2017))
EM1: If I am supposed to put in extra effort in my job, I need to get extra pay
EM2: External incentives such as bonuses and provisions are essential for how well I perform my job
EM3: It is important for me to have an external incentive to strive for in order to do a good job
EM4: If I had been offered better pay, I would have done a better job
**User orientation** (Jensen & Andersen, 2015)
UO1: The individual user is more important than formal rules
UO2: It gives me energy to know that I helped the user
UO3: If the user is satisfied, the job is done
**Public service motivation** (L. B. Andersen & Kjeldsen, 2013; Jensen & Andersen, 2015)
PSM1: To me, considering the welfare of others is one of the most important values
PSM2: It is difficult for me to contain my feelings when I see people in distress
PSM3: I am often reminded by daily events about how dependent we are on one another
PSM4: I unselfishly contribute to my community
PSM5: Meaningful public service is very important to me
PSM6: I would prefer seeing public officials do what is best for the whole community, even if it harmed my interests
PSM7: I consider public service my civic duty
PSM8: I generally associate politics with something positive
PSM9: The give and take of public policy making does not appeal to me
PSM10: I do not care much for politicians
PSM11: Making a difference in society means more to me than personal achievements
PSM12: I feel people should give back to society more than they get from it
PSM13: I am prepared to make sacrifices for the good of society
PSM14: People like me are willing to risk personal loss to help society
PSM15: I put civic duty before self

Source: Yordanov et al. (2022). See Yordanov et al. (2022) for further information about the motivational components and survey.

Online resource 2

Table 2 Motivational components, items, factor loadings, Cronbach's alpha

Motivational components, items, source	Factor loadings	Cronbach's alpha
**Extrinsic motivation (EM)**		0.78
EM1: If I am supposed to put in extra effort in my job, I need to get extra pay	0.387	
EM2: External incentives such as bonuses and provisions are essential for how well I perform my job	0.886	
EM3: It is important for me to have an external incentive to strive for in order to do a good job	0.815	
EM4: If I had been offered better pay, I would have done a better job	0.663	
**User orientation (UO)**		0.48
UO1: The individual user is more important than formal rules	0.452	
UO2: It gives me energy to know that I helped the user	0.631	
UO3: If the user is satisfied, the job is done	0.419	
**Public service motivation (PSM)**		0.79
PSM1: To me, considering the welfare of others is one of the most important values	0.412	
PSM2: It is difficult for me to contain my feelings when I see people in distress	0.418	
PSM3: I am often reminded by daily events about how dependent we are on one another	0.485	
PSM4: I unselfishly contribute to my community	0.334	
PSM5: Meaningful public service is very important to me	0.491	
PSM6: I would prefer seeing public officials do what is best for the whole community, even if it harmed my interests	0.564	
PSM7: I consider public service my civic duty	0.680	
PSM8: I generally associate politics with something positive	0.571	
PSM9: The give and take of public policy making does not appeal to me (reversed)	0.123	
PSM10: I do not care much for politicians (reversed)	0.466	
PSM11: Making a difference in society means more to me than personal achievements	0.600	
PSM12: I feel people should give back to society more than they get from it	0.578	
PSM13: I am prepared to make sacrifices for the good of society	0.855	
PSM14: People like me are willing to risk personal loss to help society	0.868	
PSM15: I put civic duty before self	0.746	

Notes: All factor loadings are statistically significant at $p<0.001$. Share of mean imputed 'do not know/not relevant' answers: EM = 1.2%, UO = 0.5%, PSM = 3.4%. The goodness-of-fit statistics for the confirmatory factor analysis for EM (chi2 = 4.58, p = 0.032; CFI = 0.994; TLI = 0.963; RMSEA = 0.056) and PSM (chi2 = 988, p <0.010; CFI = 0.371; TLI = 0.257; RMSEA = 0.094) suggest a good fit of the proposed models. As UO consists of only three items, the model is just-identified, and we therefore do not provide any goodness-of-fit statistics for this component.

Online resource 3

Table 3 Test for representativity of our sample against the study population of general practices

Variable	Sample		Study population		p-value
	Mean	SD	Mean	SD	
**Who they serve?**					
Share of enlisted patients who are/have....					
20-59-year olds and unemployed for at least 6 months	0.008	0.004	0.008	0.004	0.002
25-59-year olds without vocational education	0.107	0.025	0.111	0.028	0.002
25-65-year olds with low disposable family income	0.122	0.046	0.131	0.053	<0.001
18-59-year olds on welfare payments	0.061	0.021	0.061	0.022	0.941
0-16-year olds in family with low educational level	0.011	0.006	0.011	0.007	0.608
Immigrants and descendants from non-Western countries	0.082	0.075	0.094	0.085	<0.001
+30-year olds who are single	0.153	0.032	0.159	0.035	<0.001
+70-year olds with a low disposable family income	0.037	0.021	0.037	0.022	0.695
Charlson's Comorbidity Index equal to 1 (ill patients)	0.037	0.009	0.037	0.010	0.081
Charlson's Comorbidity Index greater than 1 (severely ill patients)	0.050	0.013	0.050	0.014	0.934
**How many they serve?**					
List size per GP in the practice	1659	454	1718	497	0.004
Whether the practice operates with a closed list	0.653	0.476	0.676	0.468	0.271
**How they serve?**					
Services provided by the practice during regular working hours to enlisted patients…					
FFS per enlisted patient (DKK)	888	153	861	174	<0.001
Number of face-to-face consultations per enlisted patient	2.94	0.50	2.90	0.55	0.097
Prescriptions redeemed by enlisted patients…					
Costs of all prescriptions per enlisted patient (DKK)	1465	301	1455	332	0.428
Number of antibiotic prescriptions issued by the GP per enlisted patient	0.273	0.085	0.277	0.096	0.256
Share of narrow-spectrum penicillin issued by the GP to enlisted patients	0.270	0.059	0.272	0.065	0.457
**Organisational factors**					
Rural location	0.342	0.475	0.283	0.451	0.004
Capital Region of Denmark	0.272	0.445	0.372	0.483	<0.001
Region Zealand	0.132	0.339	0.138	0.345	0.708
Region of Southern Denmark	0.250	0.433	0.208	0.406	0.023
Central Denmark Region	0.277	0.448	0.211	0.408	<0.001
North Denmark Region	0.069	0.254	0.071	0.257	0.846
**Practice characteristics**					
Singlehanded practice	0.309	0.463	0.452	0.498	<0.001
Number of practices	795		1584		

Notes: The study population consists of general practices that were active in the entire year 2019. p-values estimated using two-sample mean comparison t-tests with unequal variances.

Online resource 4

Table 4 Correlation between measures of motivation

	EM	UO	PSM
EM	1.000		
UO	0.152*	1.000	
PSM	-0.080*	0.050	1.000

Note: *p<0.05.

Online resource 5

Table 5.1 The link between practice motivation and who they serve (using weights from confirmatory factor analysis)

Outcome	EM	UO	PSM
Share of enlisted patients who are/have....			
20-59-year olds and unemployed for at least 6 months	0.0001	-0.0005	0.001
	(0.001)	(0.001)	(0.001)
25-59-year olds without vocational education	-0.003	0.012	0.002
	(0.004)	(0.007)	(0.007)
25-65-year olds with low disposable family income	-0.001	0.004	0.007
	(0.008)	(0.013)	(0.012)
18-59-year olds on welfare payments	-0.004	0.008	-0.001
	(0.004)	(0.006)	(0.006)
0-16-year olds in family with low educational level	-0.001	0.003	0.0001
	(0.001)	(0.002)	(0.002)
Immigrants and descendants from non-Western countries	-0.004	-0.001	0.013
	(0.0124)	(0.020)	(0.022)
+30-year olds who are single	-0.002	0.016	0.018*
	(0.005)	(0.009)	(0.009)
+70-year olds with a low disposable family income	-0.007	0.009	-0.003
	(0.004)	(0.005)	(0.006)
Charlson's Comorbidity Index equal to 1 (ill patients)	-0.002	0.003	-0.002
	(0.001)	(0.002)	(0.002)
Charlson's Comorbidity Index greater than 1 (severely ill patients)	-0.001	0.011**	0.003
	(0.002)	(0.003)	(0.003)
Number of observations (practices)		795	

Note: This table shows estimates of regressions between practice motivation (EM, UO, PSM) and care (share of high-need patients), where EM: Financial motivation, UO: Altruism towards the patient, and PSM: Altruism towards society. Estimates are based on ordinary least square regressions with robust standard errors. Standard errors are in parentheses. *p<0.05, **p<0.01, ***p<0.001.

Table 5.2 The link between practice motivation and how many they serve (using weights from confirmatory factor analysis)

	EM		UO		PSM	
Outcome	(1)	(2)	(1)	(2)	(1)	(2)
List size per GP in the practice	140.4*	128.1	71.28	54.95	-150.4	-176.6
	(68.67)	(65.49)	(117.7)	(110.6)	(107.8)	(114.4)
Whether the practice operate with a closed list	0.963	0.844	0.478	0.805	1.193	1.024
	(0.322)	(0.302)	(0.250)	(0.465)	(0.603)	(0.582)
Number of observations (practices)			795			

Note: This table shows estimates of regressions between practice motivation (EM, UO, PSM) and care (list size per GP/closed list), where EM: Financial motivation, UO: Altruism towards the patient, and PSM: Altruism towards society. Estimates for 'list size per GP' are based on ordinary least square regressions with robust standard errors. Estimates for 'whether the practice has a closed list' are based on a logit regression with robust standard errors and are reported as odds ratios. The column numbers express the included controls: (1): no controls, (2): control for who the practices' serve (see table 1 for an overview of included variables). Standard errors are in parentheses. *p<0.05, **p<0.01, ***p<0.001.

Table 5.3 The link between practice motivation and how they serve (using weights from confirmatory factor analysis)

	EM		UO		PSM	
Outcome	(1)	(2)	(1)	(2)	(1)	(2)
FFS per enlisted patient (DKK)	65.11**	82.56***	61.47	21.77	-36.83	-10.82
	(24.84)	(20.45)	(36.82)	(31.85)	(41.06)	(37.36)
Number of face-to-face consultations per	0.093	0.156*	0.180	0.020	-0.085	-0.006
enlisted patient	(0.088)	(0.074)	(0.130)	(0.114)	(0.138)	(0.126)
Costs of all prescriptions redeemed per	-61.07	-10.69	-213.8**	15.16	-122.9	-140.8***
enlisted patient (DKK)	(52.44)	(28.44)	(77.61)	(38.95)	(76.11)	(40.93)
Number of antibiotic prescriptions issued	0.006	0.007	0.065**	0.030	-0.006	0.007
by the GP per enlisted patient	(0.015)	(0.012)	(0.021)	(0.018)	(0.022)	(0.019)
Share of narrow-spectrum penicillin issued	0.00003	0.001	0.014	0.016	0.025	0.031*
by the GP to enlisted patients	(0.010)	(0.009)	(0.015)	(0.015)	(0.016)	(0.015)
Number of observations (practices)			795			

Note: This table shows estimates of regressions between practice motivation (EM, UO, PSM) and care (services and costs per patient), where EM: Financial motivation, UO: Altruism towards the patient, and PSM: Altruism towards society. Estimates are based on ordinary least square regressions with robust standard errors. The column numbers express the included controls: (1): no controls, (2): control for who they serve and how many they serve (see table 1 for an overview of included variables). Standard errors are in parentheses. *p<0.05, **p<0.01, ***p<0.001.

Online resource 6

Table 6.1 The link between practice motivation and who they serve (excluding motivational responses 'do no know/not relevant')

Outcome	EM	UO	PSM
Share of enlisted patients who are/have....			
20-59-year olds and unemployed for at least 6 months	0.001	-0.001	0.001
	(0.001)	(0.001)	(0.001)
25-59-year olds without vocational education	-0.0005	0.014	-0.0004
	(0.005)	(0.007)	(0.007)
25-65-year olds with low disposable family income	0.005	0.005	0.003
	(0.009)	(0.014)	(0.013)
18-59-year olds on welfare payments	-0.003	0.009	-0.002
	(0.004)	(0.006)	(0.006)
0-16-year olds in family with low educational level	-0.001	0.003	-0.0002
	(0.001)	(0.002)	(0.002)
Immigrants and descendants from non-Western countries	0.003	-0.002	-0.001
	(0.014)	(0.023)	(0.023)
+30-year olds who are single	-0.001	0.019*	0.010
	(0.006)	(0.009)	(0.009)
+70-year olds with a low disposable family income	-0.010*	0.014*	-0.005
	(0.004)	(0.006)	(0.006)
Charlson's Comorbidity Index equal to 1 (ill patients)	-0.002	0.004	-0.002
	(0.001)	(0.002)	(0.002)
Charlson's Comorbidity Index greater than 1 (severely ill patients)	-0.002	0.012***	0.001
	(0.002)	(0.004)	(0.003)
Number of observations (practices)		668	

Note: This table shows estimates of regressions between practice motivation (EM, UO, PSM) and care (share of high-need patients), where EM: Financial motivation, UO: Altruism towards the patient, and PSM: Altruism towards society. Estimates are based on ordinary least square regressions with robust standard errors. Standard errors are in parentheses. *p<0.05, **p<0.01, ***p<0.001.

Table 6.2 The link between practice motivation and how many they serve (excluding motivational responses 'do not know/not relevant')

	EM		UO		PSM	
Outcome	(1)	(2)	(1)	(2)	(1)	(2)
List size per GP in the practice	167.0*	135.1	102.2	97.92	-221.5	-251.1
	(79.66)	(77.54)	(135.6)	(129.5)	(122.0)	(127.9)
Whether the practice operate with a closed list	0.936	0.809	0.428	0.759	0.859	0.788
	(0.363)	(0.338)	(0.248)	(0.495)	(0.477)	(0.486)
Number of observations (practices)			668			

Note: This table shows estimates of regressions between practice motivation (EM, UO, PSM) and care (list size per GP/closed list), where EM: Financial motivation, UO: Altruism towards the patient, and PSM: Altruism towards society. Estimates for 'list size per GP' are based on ordinary least square regressions with robust standard errors. Estimates for 'whether the practice has a closed list' are based on a logit regression with robust standard errors and are reported as odds ratios. The column numbers express the included controls: (1): no controls, (2): control for who the practices' serve (see table 1 for an overview of included variables). Standard errors are in parentheses. *p<0.05, **p<0.01, ***p<0.001.

Table 6.3 The link between practice motivation and how they serve (excluding motivational responses 'do no know/not relevant')

	EM		UO		PSM	
Outcome	(1)	(2)	(1)	(2)	(1)	(2)
FFS per enlisted patient (DKK)	53.34	78.25***	76.91	32.48	-22.26	-8.39
	(28.58)	(23.57)	(40.66)	(35.70)	(43.55)	(39.99)
Number of face-to-face consultations per	0.022	0.114	0.336*	0.146	-0.039	0.019
enlisted patient	(0.101)	(0.084)	(0.144)	(0.126)	(0.152)	(0.141)
Costs of all prescriptions redeemed per	-77.37	4.24	252.6**	14.62	-138.4	-128.4**
enlisted patient (DKK)	(61.30)	(34.38)	(82.96)	(44.29)	(84.49)	(46.01)
Number of antibiotic prescriptions issued	-0.011	0.009	0.090***	0.046*	-0.007	0.009
by the GP per enlisted patient	(0.017)	(0.014)	(0.022)	(0.019)	(0.024)	(0.020)
Share of narrow-spectrum penicillin issued	-0.003	-0.003	0.021	0.021	0.023	0.026
by the GP to enlisted patients	(0.011)	(0.010)	(0.016)	(0.016)	(0.016)	(0.015)
Number of observations (practices)			668			

Note: This table shows estimates of regressions between practice motivation (EM, UO, PSM) and care (services and costs per patient), where EM: Financial motivation, UO: Altruism towards the patient, and PSM: Altruism towards society. Estimates are based on ordinary least square regressions with robust standard errors. The column numbers express the included controls: (1): no controls, (2): control for who they serve and how many they serve (see table 1 for an overview of included variables). Standard errors are in parentheses. *p<0.05, **p<0.01, ***p<0.001.

Online resource 7

Table 7.1 The link between practice motivation and who they serve (motivational components dichotomised using a 50-percentile cut-off)

Outcome	EM	UO	PSM
Share of enlisted patients who are/have....			
20-59-year olds and unemployed for at least 6 months	0.0002	-0.0003	0.0003
	(0.0003)	(0.0003)	(0.0003)
25-59-year olds without vocational education	0.001	0.001	0.003
	(0.002)	(0.002)	(0.002)
25-65-year olds with low disposable family income	0.001	-0.003	0.003
	(0.003)	(0.003)	(0.003)
18-59-year olds on welfare payments	0.0001	-0.00002	0.001
	(0.002)	(0.002)	(0.002)
0-16-year olds in family with low educational level	-0.00004	0.0001	0.001
	(0.0004)	(0.0004)	(0.0004)
Immigrants and descendants from non-Western countries	0.0001	-0.004	0.008
	(0.005)	(0.005)	(0.005)
+30-year olds who are single	-0.002	0.004	0.006*
	(0.002)	(0.002)	(0.002)
+70-year olds with a low disposable family income	-0.003*	0.002	0.001
	(0.001)	(0.002)	(0.001)
Charlson's Comorbidity Index equal to 1 (ill patients)	-0.001	0.001	-0.00005
	(0.001)	(0.001)	(0.001)
Charlson's Comorbidity Index greater than 1 (severely ill patients)	-0.001<br>(0.001)	0.003**<br>(0.001)	0.001<br>(0.001)
Number of observations (practices)		795	

Note: This table shows estimates of regressions between practice motivation (EM, UO, PSM) and care (share of high-need patients), where EM: Financial motivation, UO: Altruism towards the patient, and PSM: Altruism towards society. Estimates are based on ordinary least square regressions with robust standard errors. Standard errors are in parentheses. *p<0.05, **p<0.01, ***p<0.001.

Table 7.2 The link between practice motivation and how many they serve (motivational components dichotomised using a 50-percentile cut-off)

	EM		UO		PSM	
Outcome	(1)	(2)	(1)	(2)	(1)	(2)
List size per GP in the practice	57.43	45.38	34.43	37.94	-34.42	-44.05
	(31.62)	(30.21)	(31.60)	(30.18)	(32.73)	(34.42)
Whether the practice operate with a closed list	0.898<br>(0.135)	0.867<br>(0.142)	0.790<br>(0.122)	0.841<br>(0.140)	0.916<br>(0.137)	0.899<br>(0.147)
Number of observations (practices)			795			

Note: This table shows estimates of regressions between practice motivation (EM, UO, PSM) and care (list size per GP/closed list), where EM: Financial motivation, UO: Altruism towards the patient, and PSM: Altruism towards society. Estimates for 'list size per GP' are based on ordinary least square regressions with robust standard errors. Estimates for 'whether the practice has a closed list' are based on a logit regression with robust standard errors and are reported as odds ratios. The column numbers express the included controls: (1): no controls, (2): control for who the practices' serve (see table 1 for an overview of included variables). Standard errors are in parentheses. *p<0.05, **p<0.01, ***p<0.001.

Table 7.3 The link between practice motivation and how they serve (motivational components dichotomised using a 50-percentile cut-off)

	EM		UO		PSM	
Outcome	(1)	(2)	(1)	(2)	(1)	(2)
FFS per enlisted patient (DKK)	29.49**	34.26***	15.83	7.96	-5.81	-0.43
	(10.82)	(9.24)	(10.95)	(9.22)	(10.86)	(9.42)
Number of face-to-face consultations per	0.045	0.062*	0.066	0.035	0.0003	0.008
enlisted patient	(0.036)	(0.031)	(0.036)	(0.031)	(0.036)	(0.031)
Costs of prescriptions issued per enlisted	-29.30	-4.36	60.12**	11.53	-3.58	-26.35*
patient (DKK)	(21.39)	(11.53)	(21.54)	(11.61)	(21.38)	(11.73)
Number of antibiotic prescriptions issued	-0.006	-0.001	0.018**	0.010*	0.003	0.003
by the GP per enlisted patient	(0.006)	(0.005)	(0.006)	(0.005)	(0.006)	(0.005)
Share of narrow-spectrum penicillin issued	0.001	0.0002	0.006	0.006	0.006	0.008*
by the GP to enlisted patients	(0.004)	(0.004)	(0.004)	(0.004)	(0.004)	(0.004)
Number of observations (practices)			795			

Note: This table shows estimates of regressions between practice motivation (EM, UO, PSM) and care (services and costs per patient), where EM: Financial motivation, UO: Altruism towards the patient, and PSM: Altruism towards society. Estimates are based on ordinary least square regressions with robust standard errors. The column numbers express the included controls: (1): no controls, (2): control for who they serve and how many they serve (see table 1 for an overview of included variables). Standard errors are in parentheses. *p<0.05, **p<0.01, ***p<0.001.

Online resource 8

Table 8.1 The link between practice motivation and who they serve (motivational components dichotomised using a 75-percentile cut-off)

Outcome	EM	UO	PSM
Share of enlisted patients who are/have....			
20-59-year olds and unemployed for at least 6 months	0.0003	0.0002	0.001*
	(0.0003)	(0.0003)	(0.0003)
25-59-year olds without vocational education	0.001	0.005**	0.003
	(0.002)	(0.002)	(0.002)
25-65-year olds with low disposable family income	0.002	0.008*	0.008*
	(0.004)	(0.004)	(0.004)
18-59-year olds on welfare payments	-0.003	0.004*	-0.001
	(0.002)	(0.002)	(0.002)
0-16-year olds in family with low educational level	-0.0003	0.001*	0.0002
	(0.001)	(0.0005)	(0.001)
Immigrants and descendants from non-Western countries	0.006	0.008	0.017*
	(0.006)	(0.006)	(0.007)
+30-year olds who are single	-0.001	0.006*	0.005
	(0.003)	(0.002)	(0.003)
+70-year olds with a low disposable family income	-0.003	0.002	-0.001
	(0.002)	(0.002)	(0.002)
Charlson's Comorbidity Index equal to 1 (ill patients)	-0.0004	0.001	-0.001
	(0.001)	(0.001)	(0.001)
Charlson's Comorbidity Index greater than 1 (severely ill patients)	-0.001	0.002*	0.0001
	(0.001)	(0.001)	(0.001)
Number of observations (practices)		795	

Note: This table shows estimates of regressions between practice motivation (EM, UO, PSM) and care (share of high-need patients), where EM: Financial motivation, UO: Altruism towards the patient, and PSM: Altruism towards society. Estimates are based on ordinary least square regressions with robust standard errors. Standard errors are in parentheses. *p<0.05, **p<0.01, ***p<0.001.

Table 8.2 The link between practice motivation and how many they serve (motivational components dichotomised using a 75-percentile cut-off)

	EM		UO		PSM	
Outcome	(1)	(2)	(1)	(2)	(1)	(2)
List size per GP in the practice	94.50*	80.95*	67.45	56.52	-13.30	-26.56
	(36.89)	(36.16)	(37.95)	(34.43)	(33.18)	(32.43)
Whether the practice operate with a closed list	1.062	1.000	0.825	0.933	1.108	1.006
	(0.185)	(0.188)	(0.128)	(0.161)	(0.193)	(0.191)
Number of observations (practices)			795			

Note: This table shows estimates of regressions between practice motivation (EM, UO, PSM) and care (list size per GP/closed list), where EM: Financial motivation, UO: Altruism towards the patient, and PSM: Altruism towards society. Estimates for 'list size per GP' are based on ordinary least square regressions with robust standard errors. Estimates for 'whether the practice has a closed list' are based on a logit regression with robust standard errors and are reported as odds ratios. The column numbers express the included controls: (1): no controls, (2): control for who the practices' serve (see table 1 for an overview of included variables). Standard errors are in parentheses. *p<0.05, **p<0.01, ***p<0.001.

Table 8.3 The link between practice motivation and how they serve (motivational components dichotomised using a 75-percentile cut-off)

	EM		UO		PSM	
Outcome	(1)	(2)	(1)	(2)	(1)	(2)
FFS per enlisted patient (DKK)	21.75	37.20***	5.94	-0.68	-23.74	-3.60
	(13.06)	(10.89)	(11.49)	(10.09)	(12.95)	(11.14)
Number of face-to-face consultations per	0.018	0.061	0.044	0.013	-0.051	0.002
enlisted patient	(0.043)	(0.035)	(0.037)	(0.033)	(0.043)	(0.037)
Costs of all prescriptions redeemed per	-9.42	19.16	59.48**	21.19	-41.68	-22.35
enlisted patient (DKK)	(24.79)	(12.94)	(22.92)	(12.21)	(25.88)	(14.60)
Number of antibiotic prescriptions issued	0.005	0.010	0.021**	0.015**	-0.005	0.002
by the GP per enlisted patient	(0.008)	(0.006)	(0.007)	(0.006)	(0.008)	(0.006)
Share of narrow-spectrum penicillin issued	-0.002	-0.002	0.003	0.004	0.003	0.005
by the GP to enlisted patients	(0.005)	(0.004)	(0.005)	(0.004)	(0.005)	(0.005)
Number of observations (practices)			795			

Note: This table shows estimates of regressions between practice motivation (EM, UO, PSM) and care (services and costs per patient), where EM: Financial motivation, UO: Altruism towards the patient, and PSM: Altruism towards society. Estimates are based on ordinary least square regressions with robust standard errors. The column numbers express the included controls: (1): no controls, (2): control for who they serve and how many they serve (see table 1 for an overview of included variables). Standard errors are in parentheses. *p<0.05, **p<0.01, ***p<0.001.

Online resource 9

Table 9.1 The link between practice motivation and who they serve (only singlehanded practices)

Outcome	EM	UO	PSM
Share of enlisted patients who are/have....			
20-59-year olds and unemployed for at least 6 months	-0.003	-0.0004	0.002
	(0.001)	(0.002)	(0.002)
25-59-year olds without vocational education	-0.014	0.024	0.005
	(0.009)	(0.013)	(0.012)
25-65-year olds with low disposable family income	-0.023	0.015	0.012
	(0.016)	(0.025)	(0.022)
18-59-year olds on welfare payments	-0.007	0.020	-0.001
	(0.007)	(0.012)	(0.010)
0-16-year olds in family with low educational level	-0.005*	0.008**	0.001
	(0.002)	(0.003)	(0.003)
Immigrants and descendants from non-Western countries	-0.054*	0.021	0.045
	(0.026)	(0.041)	(0.046)
+30-year olds who are single	-0.003	0.010	0.018
	(0.009)	(0.014)	(0.014)
+70-year olds with a low disposable family income	-0.004	0.012	-0.010
	(0.007)	(0.010)	(0.010)
Charlson's Comorbidity Index equal to 1 (ill patients)	-0.001	0.004	-0.005
	(0.002)	(0.004)	(0.003)
Charlson's Comorbidity Index greater than 1 (severely ill patients)	0.003<br>(0.004)	0.013*<br>(0.006)	0.001<br>(0.005)
Number of observations (practices)		246	

Note: This table shows estimates of regressions between GP motivation (EM, UO, PSM) and practice care (share of high-need patients), where EM: Financial motivation, UO: Altruism towards the patient, and PSM: Altruism towards society. Estimates are based on ordinary least square regressions with robust standard errors. Standard errors are in parentheses. *p<0.05, **p<0.01, ***p<0.001.

Table 9.2 The link between practice motivation and how many they serve (only singlehanded practices)

	EM		UO		PSM	
Outcome	(1)	(2)	(1)	(2)	(1)	(2)
List size per GP in the practice	112.7	125.1	164.8	71.15	-338.3	-294.2
	(124.3)	(119.7)	(239.8)	(204.2)	(204.5)	(223.8)
Whether the practice operate with a closed list	1.068<br>(0.656)	1.133<br>(0.802)	0.236<br>(0.224)	0.805<br>(0.814)	0.418<br>(0.379)	0.390<br>(0.402)
Number of observations (practices)			246			

Note: This table shows estimates of regressions between GP motivation (EM, UO, PSM) and practice care (list size per GP/closed list), where EM: Financial motivation, UO: Altruism towards the patient, and PSM: Altruism towards society. Estimates for 'list size per GP' are based on ordinary least square regressions with robust standard errors. Estimates for 'whether the practice has a closed list' are based on a logit regression with robust standard errors and are reported as odds ratios. The column numbers express the included controls: (1): no controls, (2): control for who the practices' serve (see table 1 for an overview of included variables). Standard errors are in parentheses. *p<0.05, **p<0.01, ***p<0.001.

Table 9.3 The link between practice motivation and how they serve (only singlehanded practices)

	EM		UO		PSM	
Outcome	(1)	(2)	(1)	(2)	(1)	(2)
FFS per enlisted patient (DKK)	151.4**	135.7***	71.63	-21.08	-76.40	-12.21
	(45.92)	(36.46)	(70.01)	(61.83)	(71.28)	(62.05)
Number of face-to-face consultations per	0.302	0.281*	0.249	-0.120	-0.269	-0.085
enlisted patient	(0.164)	(0.136)	(0.242)	(0.209)	(0.221)	(0.199)
Costs of all prescriptions redeemed per	18.47	-1.042	294.9	-35.09	-325.6*	-300.6***
enlisted patient (DKK)	(98.82)	(53.96)	(150.7)	(74.22)	(133.6)	(72.92)
Number of antibiotic prescriptions issued	0.029	0.035	0.080	0.013	-0.012	0.026
by the GP per enlisted patient	(0.029)	(0.026)	(0.041)	(0.037)	(0.039)	(0.035)
Share of narrow-spectrum penicillin issued	-0.018	-0.002	0.012	0.011	0.020	0.022
by the GP to enlisted patients	(0.019)	(0.018)	(0.029)	(0.030)	(0.030)	(0.029)
Number of observations (practices)			246			

Note: This table shows estimates of regressions between GP motivation (EM, UO, PSM) and practice care (services and costs per patient), where EM: Financial motivation, UO: Altruism towards the patient, and PSM: Altruism towards society. Estimates are based on ordinary least square regressions with robust standard errors. The column numbers express the included controls: (1): no controls, (2): control for who they serve and how many they serve (see table 1 for an overview of included variables). Standard errors are in parentheses. *p<0.05, **p<0.01, ***p<0.001.

Online resource 10

Table 10.1 The link between practice motivation and who they serve (controlling for structural factors)

Outcome	EM	UO	PSM
Share of enlisted patients who are/have....			
20-59-year olds and unemployed for at least 6 months	0.0002	-0.0001	0.001
	(0.001)	(0.001)	(0.001)
25-59-year olds without vocational education	-0.002	0.011	0.002
	(0.005)	(0.007)	(0.007)
25-65-year olds with low disposable family income	-0.00005	0.004	0.010
	(0.008)	(0.012)	(0.012)
18-59-year olds on welfare payments	-0.001	0.005	0.002
	(0.003)	(0.005)	(0.005)
0-16-year olds in family with low educational level	-0.001	0.002	0.001
	(0.001)	(0.002)	(0.002)
Immigrants and descendants from non-Western countries	-0.008	0.004	0.014
	(0.012)	(0.018)	(0.022)
+30-year olds who are single	-0.001	0.018*	0.017
	(0.005)	(0.008)	(0.009)
+70-year olds with a low disposable family income	-0.005	0.008	-0.001
	(0.003)	(0.005)	(0.005)
Charlson's Comorbidity Index equal to 1 (ill patients)	-0.002	0.002	-0.003
	(0.001)	(0.002)	(0.002)
Charlson's Comorbidity Index greater than 1 (severely ill patients)	-0.001	0.008**	0.0004
	(0.002)	(0.003)	(0.003)
Number of observations (practices)		795	

Note: This table shows estimates of regressions between practice motivation (EM, UO, PSM) and care (share of high-need patients), where EM: Financial motivation, UO: Altruism towards the patient, and PSM: Altruism towards society. Estimates are based on ordinary least square regressions with robust standard errors. Standard errors are in parentheses. *p<0.05, **p<0.01, ***p<0.001.

Table 10.2 The link between practice motivation and how many they serve (controlling for structural factors)

	EM		UO		PSM	
Outcome	(1)	(2)	(1)	(2)	(1)	(2)
List size per GP in the practice	141.2*	136.0*	119.6	112.0	-192.7	-208.8
	(71.78)	(68.88)	(115.1)	(112.4)	(120.5)	(126.9)
Whether the practice operate with a closed list	0.994	0.963	0.638	0.933	1.273	1.203
	(0.385)	(0.385)	(0.357)	(0.552)	(0.735)	(0.739)
Number of observations (practices)			795			

Note: This table shows estimates of regressions between practice motivation (EM, UO, PSM) and care (list size per GP/closed list), where EM: Financial motivation, UO: Altruism towards the patient, and PSM: Altruism towards society. Estimates for 'list size per GP' are based on ordinary least square regressions with robust standard errors. Estimates for 'whether the practice has a closed list' are based on a logit regression with robust standard errors and are reported as odds ratios. The column numbers express the included controls: (1): no controls, (2): control for who the practices' serve (see table 1 for an overview of included variables). Standard errors are in parentheses. *p<0.05, **p<0.01, ***p<0.001.

Table 10.3 The link between practice motivation and how they serve (controlling for structural factors)

	EM		UO		PSM	
Outcome	(1)	(2)	(1)	(2)	(1)	(2)
FFS per enlisted patient (DKK)	84.79***	86.68***	36.70	6.76	-15.65	6.94
	(23.58)	(21.02)	(32.44)	(30.69)	(39.07)	(36.49)
Number of face-to-face consultations per	0.132	0.152	0.159	0.045	-0.019	0.056
enlisted patient	(0.086)	(0.078)	(0.122)	(0.112)	(0.136)	(0.127)
Costs of all prescriptions redeemed per	-52.35	-15.74	219.1**	48.21	-129.4	-122.8**
enlisted patient (DKK)	(51.44)	(29.69)	(69.06)	(36.09)	(72.92)	(41.60)
Number of antibiotic prescriptions issued	-0.003	0.006	0.066***	0.039*	-0.006	0.012
by the GP per enlisted patient	(0.015)	(0.013)	(0.019)	(0.017)	(0.022)	(0.019)
Share of narrow-spectrum penicillin issued	0.001	0.002	0.003	0.012	0.021	0.026
by the GP to enlisted patients	(0.010)	(0.010)	(0.014)	(0.014)	(0.016)	(0.015)
Number of observations (practices)			795			

Note: This table shows estimates of regressions between practice motivation (EM, UO, PSM) and care (services and costs per patient), where EM: Financial motivation, UO: Altruism towards the patient, and PSM: Altruism towards society. Estimates are based on ordinary least square regressions with robust standard errors. The column numbers express the included controls: (1): no controls, (2): control for who they serve and how many they serve (see table 1 for an overview of included variables). Standard errors are in parentheses. *p<0.05, **p<0.01, ***p<0.001.
